# REC-1 and HIM-5 distribute meiotic crossovers and function redundantly in meiotic double-strand break formation in *Caenorhabditis elegans*

**DOI:** 10.1101/gad.266056.115

**Published:** 2015-09-15

**Authors:** George Chung, Ann M. Rose, Mark I.R. Petalcorin, Julie S. Martin, Zebulin Kessler, Luis Sanchez-Pulido, Chris P. Ponting, Judith L. Yanowitz, Simon J. Boulton

**Affiliations:** 1 Department of Medical Genetics, University of British Columbia, Vancouver, British Columbia V6T 1Z4, Canada;; 2 DNA Damage Response Laboratory, The Francis Crick Institute, South Mimms EN3 3LD, United Kingdom;; 3 Clare Hall Laboratories, The Francis Crick Institute, South Mimms EN3 3LD, United Kingdom;; 4 Magee-Womens Research Institute, Department of Obstetrics, Gynecology, and Reproductive Sciences, University of Pittsburgh School of Medicine, Pittsburgh, Pennsylvania 15213, USA;; 5 Medical Research Council Functional Genomics Unit, Department of Physiology, Anatomy, and Genetics, University of Oxford, Oxford OX1 3PT, United Kingdom

**Keywords:** meiosis, meiotic crossover distribution, REC-1, HIM-5, cyclin-dependent kinase

## Abstract

The *Caenorhabditis elegans* gene *rec-1* was the first genetic locus identified in metazoa to affect the distribution of meiotic crossovers along the chromosome. We report that *rec-1* encodes a distant paralog of HIM-5, which was discovered by whole-genome sequencing and confirmed by multiple genome-edited alleles. REC-1 is phosphorylated by cyclin-dependent kinase (CDK) in vitro, and mutation of the CDK consensus sites in REC-1 compromises meiotic crossover distribution in vivo. Unexpectedly, *rec-1; him-5* double mutants are synthetic-lethal due to a defect in meiotic double-strand break formation. Thus, we uncovered an unexpected robustness to meiotic DSB formation and crossover positioning that is executed by HIM-5 and REC-1 and regulated by phosphorylation.

## Abstract (precis)

In this study, Chung et al. use whole-genome sequencing to demonstrate that *rec-1* encodes a distant paralog of HIM-5. They also show that REC-1 is phosphorylated by CDK in vitro, and mutation of the CDK consensus sites in REC-1 compromises meiotic crossover distribution in vivo. These findings provide new insights into meiotic DSB formation and crossover positioning.

DNA double-strand breaks (DSBs) are one of the most deleterious lesions to our genome yet are induced during meiosis to promote the exchange of genetic material between homologous chromosomes. Accordingly, generation of meiotic DSBs is tightly regulated by kinases and is coordinated with cell cycle progression to ensure their proper timing and repair in order to generate meiotic crossover events (Baudat et al. 2013; de Massy 2013; Lui and Colaiácovo 2013; Murakami and Keeney 2014). Although meiotic crossover events are distributed nonrandomly along the chromosome in many taxa (Brenner 1974; Kaback et al. 1989; Oliver et al. 1992; Kliman and Hey 1993; Barnes et al. 1995; Nachman and Churchill 1996; Yu et al. 2001; Solignac et al. 2007; Giraut et al. 2011; Ross et al. 2011), the precise mechanism by which crossover distribution patterns are established remains poorly understood.

In the self-fertilizing hermaphrodite *Caenorhabditis elegans*, autosomes have highly recombinogenic arms flanking lowly recombinogenic centers (Barnes et al. 1995). Moreover, crossover interference across the autosomes is almost complete, resulting in a single crossover event per homolog pair in most meioses (Hodgkin et al. 1979; Zetka and Rose 1995; Meneely et al. 2002; Hillers and Villeneuve 2003; Nabeshima et al. 2004; Lim et al. 2008; Gabdank and Fire 2014), which also facilitates the determination of the crossover locations. A recessive mutation in the *rec-1* gene randomizes the distribution of the meiotic recombination events while preserving crossover interference such that an increased crossover frequency in the autosomal centers is accompanied by a decreased crossover frequency in the autosomal arms (Rose and Baillie 1979; Zetka and Rose 1995). While the total number of crossover events remains unaltered at one per homolog pair, their positions differ dramatically between mutant *rec-1* and wild type. Notably, *rec-1* was the first locus described in *C. elegans* to exert such genetic control of the meiotic crossover pattern without perturbing crossover interference. Because of this altered recombination phenotype and the absence of any additional effects on development or fecundity (Rattray and Rose 1988), mapping of *rec-1* by conventional linkage analysis was not possible, and the identity of *C. elegans rec-1* remained unknown for >30 years after the description of its mutant phenotype. This is in contrast to several other *C. elegans* loci that, while exerting a genetic control over the meiotic crossover distribution similar to that of *rec-1*, were initially identified on the basis of severe meiotic nondisjunction phenotypes, such as *xnd-1* (Wagner et al. 2010) and *him-5* (Hodgkin et al. 1979; Meneely et al. 2002, 2012), or a priori knowledge of the gene product or function, such as *slx-1* (Saito et al. 2012, 2013).

In this study, we set out to determine the molecular identity of the *rec-1* gene using whole-genome sequencing data (Rose et al. 2010) and generate putative *rec-1* alleles using genome-editing techniques in *C. elegans*. We show that the REC-1 protein is a distant paralog of HIM-5 and is a substrate for phosphorylation by cyclin-dependent kinase (CDK) in vitro. Genetic analysis of a REC-1 phospho-mutant transgene revealed a critical role for phosphorylation in patterning meiotic crossovers in vivo. We also establish an unappreciated redundancy in meiotic DSB formation based on a significant reduction in meiotic DSBs in *rec-1; him-*5 double mutants. Thus, our data highlight an evolutionary and functional relationship between *rec-1* and *him-5* in the generation of meiotic DSBs and their distribution on meiotic chromosomes.

## Results

### The molecular identity of *rec-1* is sequence *y18h1a.7* on chromosome I

Despite the difficulties of scoring a second-generation crossover phenotype, genetic mapping positioned the *rec-1* gene to an interval in chromosome I (NJ O'Neil and AM Rose, unpubl.). The genomic sequence of a strain carrying the *rec-1(s180)* mutation contained 441 single-nucleotide differences when compared with the wild-type progenitor (Rose et al. 2010). Using the map position of the *rec-1(s180)* mutation and the DNA sequence information, a nonsense mutation affecting the coding sequence *y18h1a.7* was identified (JSC Chu and AM Rose, unpubl.). RNAi knockdown of *y18h1a.7* resulted in an altered distribution of crossover events that partly recapitulated the Rec-1 phenotype (J Luce, M Jones, and AM Rose, unpubl.).

**Figure 1.**
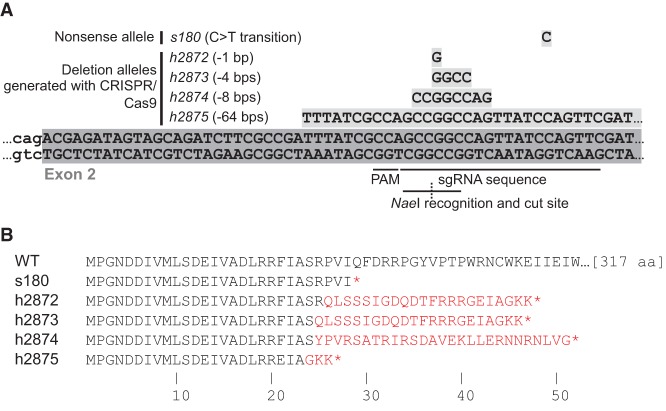
The *C. elegans rec-1* mutations map to *y18h1a.7*. (*A*) The *s180* allele was identified by a whole-genome sequencing experiment described previously (Rose et al. 2010). Four alleles of *rec-1* were generated by CRISPR–Cas9 (Friedland et al. 2013) using the same target guide RNA sequence and the protospacer-adjacent motif (PAM). (*B*) The mutant alleles of *rec-1* encode truncated versions of REC-1. Amino acid differences from the wild-type translation are colored in red.

**Figure 2.**
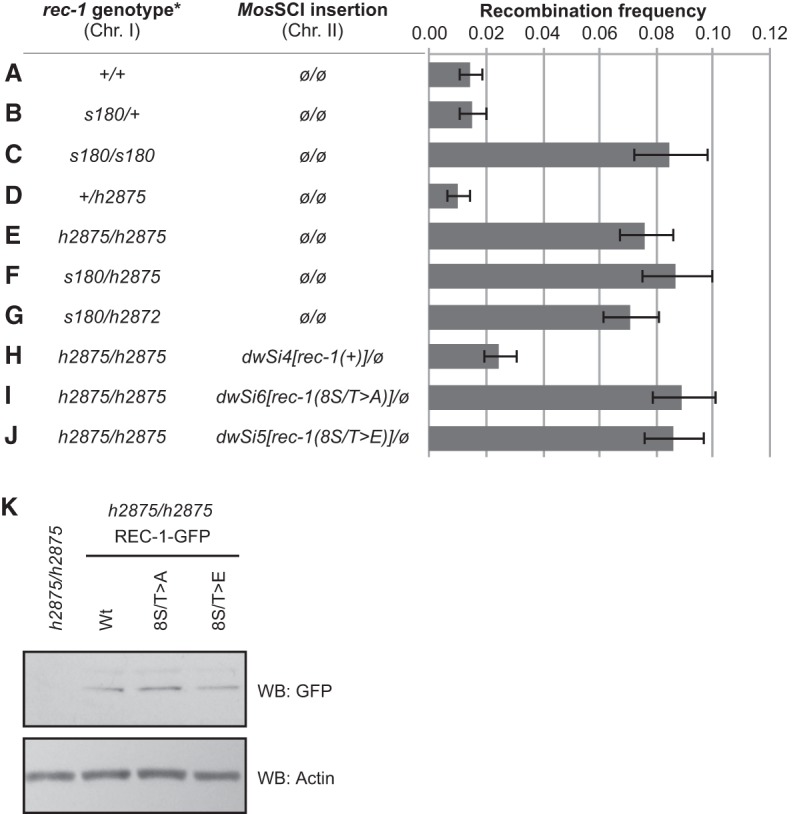
Mutations in *rec-1* cause an increased recombination frequency in the *dpy-5–unc-13* genetic interval. (*A–C*) The *s180* allele of *rec-1* confers a recessive increase of recombination frequency in this interval. (*D*,*E*) The *h2875* allele of *y18h1a.7* also confers a recessive increase of recombination frequency in this interval. (*F*,*G*) The *h2875* and *h2872* alleles of *y18h1a.7* fail to complement *rec-1(s180)*. (*H*) The reintroduction of wild-type *rec-1* by *Mos*SCI reverses this increase in recombination frequency. (*I*,*J*) The insertion of *rec-1* mutated at putative phosphorylation sites fails to rescue the altered recombination phenotype. See the text for a description of the alleles. (ø) The absence of a *Mos-1-*mediated transgene insertion. (*) Where applicable, the first allele indicates the homolog bearing the *dpy-5* and *unc-13* mutations. Error bars indicate the 95% Copper-Pearson confidence interval. (*K*) C-terminal, GFP-tagged versions of REC-1 expressed from the same integration site as other *rec-1* integration alleles were probed. The comparably expressed bands suggest that promoter activity was adequate and that translation products were stable despite replaced S/T residues.

In order to confirm that *y18h1a.7* is the coding region whose mutation confers the Rec-1 phenotype, we targeted the transgenic Cas9 enzyme (Friedland et al. 2013) to cut the second exon of the gene—the same exon predicted to be disrupted by the nonsense mutation in *rec-1(s180)*. From this, we generated four frameshift deletion alleles of *y18h1a.7* (Fig. 1A). The largest deletion, *h2875*, conferred a recessive increase of recombination frequency in the *dpy-5–unc-13* genetic interval, as had been described for *s180* (Fig. 2A–E; Zetka and Rose 1995). In addition, *h2875* failed to complement *s180* with respect to the recombination frequency in both the *dpy-5–unc-13* and *unc-101–unc-54* intervals (Fig. 2F; Supplemental Fig. 1). The smaller deletion, *h2872*, also failed to complement *s180* with respect to the frequency of recombination in the *dpy-5–unc-13* interval (Fig. 2G). Furthermore, a single wild-type copy of *y18h1a.7* {*dwSi4[rec-1(+)]*} inserted into chromosome II via *Mos1*-mediated single-copy insertion (*Mos*SCI) (Frøkjær-Jensen et al. 2008) restored the wild-type frequency of recombination events in the *dpy-5–unc-13* interval (Fig. 2H). Similar to observations of *rec-1(s180)* homozygotes, which appear to maintain strict crossover interference (Zetka and Rose 1995), we found no evidence of double crossovers in the oocytes of *rec-1(h2875)* homozygotes (Supplemental Fig. 2). Like *rec-1(s180)* homozygotes (Rattray and Rose 1988), *rec-1(h2875)* homozygotes also had a mild increase in the number of spontaneous male progeny (0.35%, *N* = 3118) compared with wild type (0.05%, *N* = 2574). This suggested that REC-1 may be involved in proper disjunction of the X chromosome but not of the autosomes, since the overall embryonic hatching frequency was unchanged from wild type (Fig. 5A, below). Collectively, these results establish that the altered recombination phenotype is caused by disruption of the gene encoded by *y18h1a.7*.

### Phosphorylatable residues in REC-1 are required for proper function

**Figure 3.**
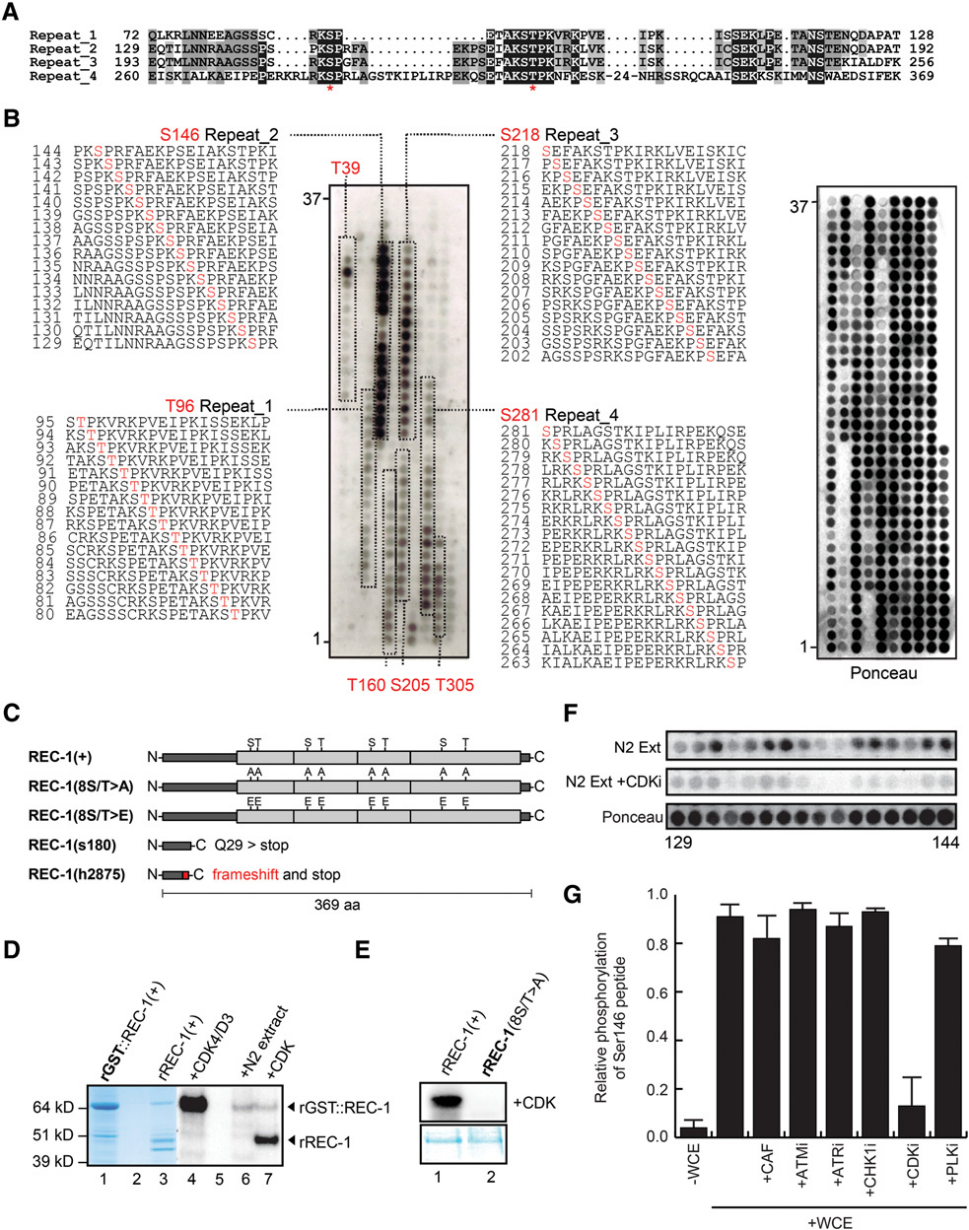
REC-1 is phosphorylated in vitro by recombinant CDK4/cyclin D3. (*A*) Alignment of the four repeats (Repeat_1 to Repeat_4) of REC-1 and the position of putative CDK phosphorylation sites (red asterisk). Identical residues are highlighted in black, and regions of similarity are highlighted in gray. (*B*) In vitro phosphorylation of the REC-1 peptide array by CDK4/D3. Each of the 350 spots represents a 20-mer peptide fragment juxtaposed by one amino acid (aa) scanning the complete REC-1 protein. Each peptide has a 19-amino-acid overlap with the previous peptide and is numbered sequentially from the start codon. Positive serial spots (detected by autoradiography) corresponding to the phosphorylated repeat regions are boxed and labeled. The peptide sequences of Repeat_2 and Repeat_3 show strong phosphorylation, with the residues S218 and S146 (highlighted in red) as possible phosphorylation sites. Stochastic variations in phosphorylation signals and peptide amount (shown by Ponceau staining) are present on the array. (*C*) Schematic representation of wild-type [REC-1(+)], phospho-mutant [REC-1(8S/T > A)], phospho-mimetic [REC-1(8S/T > E)] and truncated [REC-1(s180) and REC-1(h2875)] versions of proteins showing relative sites of the eight-residue substitutions or the deleted regions. (*D*) The GST-REC-1 fusion protein [rGST::REC-1(+)], shown as 64-kDa Coomassie-stained band in lane *1*, is phosphorylated in vitro by CDK4/D3 (lanes *4*,*7*) and N2 wild-type extract (lane *6*). Lanes *2* and *5* are blanks. Lanes *3*, *6*, and *7* are loaded with recombinant REC-1 protein, and most of the recombinant protein in lane *7* had the GST moiety removed by PreScission protease cleavage. REC-1 without the GST moiety is shown as 44-kDa Coomassie-stained band (lane *3*) that is phosphorylated by CDK4/D3 (lane *7*). (Lanes *6*,*7*) Remnants of the uncleaved full-length fusion protein rGST::REC-1 (64 kDa) are shown as phosphorylated bands by both wild-type extract and CDK4/D3. (*E*) Phosphorylation of recombinant wild-type REC-1 and the absence of phosphorylation of phospho-mutant REC-1 (8S/T > A) by CDK4/D3, shown with the corresponding Coomassie-stained gel. (*F*) Comparative in vitro phosphorylation of the phosphorylated Repeat_3-containing sequential peptides 129–144 by N2 worm extract (*top*) and N2 worm extract containing the CDK inhibitor (CDKi) roscovitine (*middle*). (*Bottom*) Ponceau staining of the peptide array membrane used is included. (*G*) Biotinylated peptide corresponding to sequence 140 from *B*, which contains Ser146, was subjected to phosphorylation in *C. elegans* N2 extracts (whole-cell extracts [WCEs]), and this phosphorylation was unaffected by caffeine or inhibitors of ATM, ATR, Chk1, or PLK1 kinases (5 µM). In contrast, phosphorylation of this peptide was substantially reduced in *C. elegans* N2 extracts (WCE) supplemented with 5 µM CDKi. Error bars represent standard deviations calculated from three separate experiments.

The predicted amino acid sequence of REC-1 contains four copies of a sequence, each containing two consensus CDK phosphorylation motifs: S/T-P (Fig. 3A). To ascertain whether CDK is able to phosphorylate REC-1 in vitro, we first assayed for CDK phosphorylation using peptide arrays. Peptides containing the S/T-P motif from each of the four repeats were phosphorylated by recombinant CDK4/cyclin D3 in vitro, with Ser146 within repeat 2 being the most heavily phosphorylated (Fig. 3B). To determine whether the S/T-P motifs are a substrate for CDK phosphorylation within full-length REC-1, we generated a mutant REC-1 in which all eight serine/threonine residues were replaced with alanines (8S/T > A) and expressed both this mutant and the wild-type protein in insect cells as a glutathione S-transferase (GST) fusion (Fig. 3C). Despite substantial CDK phosphorylation of the wild-type GST-REC-1 (Fig. 3D, lane 4) and REC-1 following removal of the GST tag (Fig. 3D, lane 7), the purified 8S/T > A mutant REC-1 could not be phosphorylated by CDK4/cyclin D3 in vitro (Fig. 3E).

While the putative phosphorylation sites in REC-1 fit the CDK consensus S/T-P and could be phosphorylated in vitro by recombinant CDK4, we wished to further investigate whether these sites are phosphorylated in *C. elegans* extracts and whether the kinase responsible is a CDK. In support of a role for a CDK in phosphorylating REC-1 in vivo, peptides containing Ser146 from repeat 2 were readily phosphorylated in *C. elegans* N2 extracts but not extracts supplemented with the CDK inhibitor (CDKi) roscovitine (Fig. 3F). Furthermore, a biotinylated peptide corresponding to sequence 140 (Fig. 3B), which contains Ser146, is phosphorylated in *C. elegans* N2 extracts (whole-cell extracts [WCE]), and this phosphorylation is unaffected by caffeine or inhibitors of ATM, ATR, Chk1, or PLK1 kinases. In contrast, phosphorylation of the Ser146-containing peptide is substantially reduced in *C. elegans* N2 extracts supplemented with roscovitine (CDKi). These results establish that REC-1 can be phosphorylated in vitro by recombinant CDK4/cyclin D3 and also in *C. elegans* extracts by a kinase that is inhibited by roscovitine, which is most likely a CDK.

To test whether the pattern of meiotic recombination events in *C. elegans* is affected by mutating the S/T-P motifs in REC-1, we integrated mutated transgenic alleles of *rec-1* into the *ttTi5605* site on chromosome II. One construct, *dwSi6[rec-1(8S/T* > *A)]*, replaced the eight serine/threonine codons with alanine codons and encoded a protein product that could not be phosphorylated by CDK4/cyclin D3 in vitro (Fig. 3C,E). A second construct, *dwSi5[rec-1(8S/T* > *E)]*, replaced the eight serine/threonine codons with glutamic acid codons and encoded a form of REC-1 mimicking constitutive phosphorylation at these eight S/T-P motifs (Fig. 3C,E). In contrast to the rescuing wild-type transgene *dwSi4[rec-1(+)]*, neither the *dwSi6[rec-1(8S/T* > *A)]* nor the *dwSi5[rec-1(8S/T* > *E)]* allele was able to rescue the altered recombination phenotype of *rec-1(h2875)* (Fig. 2H–J). Integrated, C-terminal GFP-tagged versions of *rec-1(+)*, *rec-1(S/T* > *A)*, and *rec-1(S/T* > *E)* can be detected by anti-GFP antisera at comparable levels regardless of the S/T amino acid changes (Fig. 2K), which establishes that the expression of integrated mutant *rec-1(S/T* > *A)* and *rec-1(S/T* > *E)* is unaffected by these amino acid changes. However, attempts to detect the REC-1-GFP fusions in the germline by immunofluorescence were unsuccessful, suggesting that the normal levels of REC-1 expression are very low, which prohibited further studies of the germline or subcellular localization of REC-1. Nevertheless, these results establish that the eight S/T-P motifs within REC-1 are important for establishing the normal pattern of meiotic recombination events in *C. elegans.* Since the transgene containing the phospho-mimetic changes within the eight S/T-P motifs was also unable to rescue the Rec-1 phenotype, it is possible that dephosphorylation of these sites is also important for REC-1 function in vivo.

### The *rec-1* gene is a distantly related paralog of *HIM-5*

**Figure 4.**
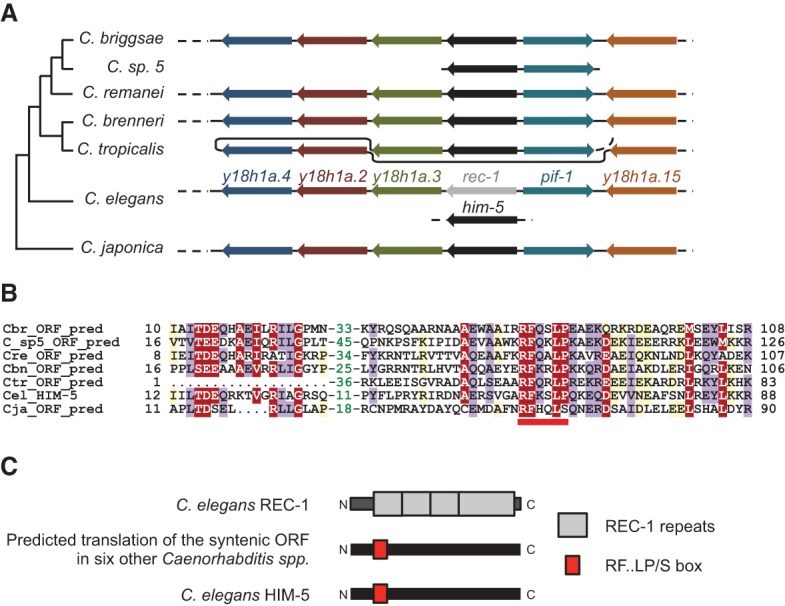
Phylogenetic analysis of *rec-1* and the surrounding synteny block reveals the identity of *rec-1* homologs. (*A*) The synteny block that contains *rec-1* appears to be conserved in six other *Caenorhabditis* species. Gene order and relative orientation are shown but not to scale. The putative ORF between the *y18h1a.3* and *pif-1* orthologs has a higher degree of sequence similarity to *C. elegans him-5* (indicated in black) than it has to *rec-1* (indicated in gray). The species are grouped on a phylogenetic tree suggested by Félix et al. (2014). ORFs with sequence similarity are depicted with the same color. (*B*) Multiple alignments of the N-terminal regions of ORFs are shown in gray. (Cel_ HIM-5) *C. elegans* HIM-5 sequence; (Cbr_ORF_pred) orthologs in *C. briggsae*; (C_sp5_ORF_pred) orthologs in *Caenorhabditis* sp. 5; (Cre_ORF_pred) orthologs in *C. remanei*; (Cbn_ORF_pred) orthologs in *C. brenneri*; (Ctr_ORF_pred) orthologs in *C. tropicalis*; (Cja_ ORF_pred) orthologs in *C. japonica*. The motif R-F-x-x-L-P/S is underlined in red. The alignment was presented with the program Belvu (Sonnhammer and Hollich 2005) using a coloring scheme indicating the average BLOSUM62 scores (which are correlated with amino acid conservation) of each alignment column: >2 in red, between 2 and 1 in violet, and between 1 and 0.3 in light yellow. Numbers shown in green represent amino acids that have been removed from the alignment. (*C*) The relative positions of motifs (the REC-1 repeat motifs and the RF..LP/S box) within the translation products of *rec-1* homologs.

Phylogenetic analysis revealed that *rec-1* resides in a synteny block of genes on chromosome I that is conserved in order and orientation and is shared among at least six other *Caenorhabditis* species: *Caenorhabditis briggsae*, *Caenorhabditis remanei*, *Caenorhabditis brenneri*, *Caenorhabditis sinica* (sp. 5), *Caenorhabditis tropicalis*, and *Caenorhabditis japonica* (Fig. 4A). Within the synteny blocks is a coding region of a size similar to and in the same orientation and position as that of the *rec-1* gene in *C. elegans* (Fig. 4A, ORFs in black). Notably, the translation product of the *C. remanei* ORF contains a short stretch of sequence similar to that of *C. elegans* REC-1 at the N terminus (Supplemental Fig. 3). Although the overall sequence match to *C. elegans* REC-1 is not strong, the translation products of the ORFs in this position in the six other species have notable sequence similarity to each other (Fig. 4B), and all contain an R-F-x-x-L-P/S motif (Fig. 4B,C). Surprisingly, we found that the ORFs in this position in the other species encode proteins that all share sequence similarity with HIM-5, whose coding sequence is located on *C. elegans* chromosome V, outside the *rec-1* synteny block. HIM-5 contains the R-F-x-x-L-P/S motif that *C. elegans* REC-1 is lacking (Fig. 4B,C). Thus, in other *Caenorhabditis* species, the gene positionally equivalent to *C. elegans rec-1* shares more sequence similarity with *C. elegans him-5* than with *C. elegans rec-1.* Altogether, the similarity between *C. elegans* REC-1 and the *C. remanei* ORF translation product (Supplemental Fig. 3), the positional equivalence of *C. elegans rec-1* to the ORFs in six other *Caenorhabditis* species that are related to *him-5* (Fig. 4A), and the phenotypic similarity between *rec-1* and *him-5* mutants with respect to the distribution of crossover events (see below) (Meneely et al. 2012) strongly suggest that REC-1 is a distantly related paralog of HIM-5.

### rec-1 and him-5 exhibit synthetic embryonic lethality due to meiotic CO defects

**Figure 5.**
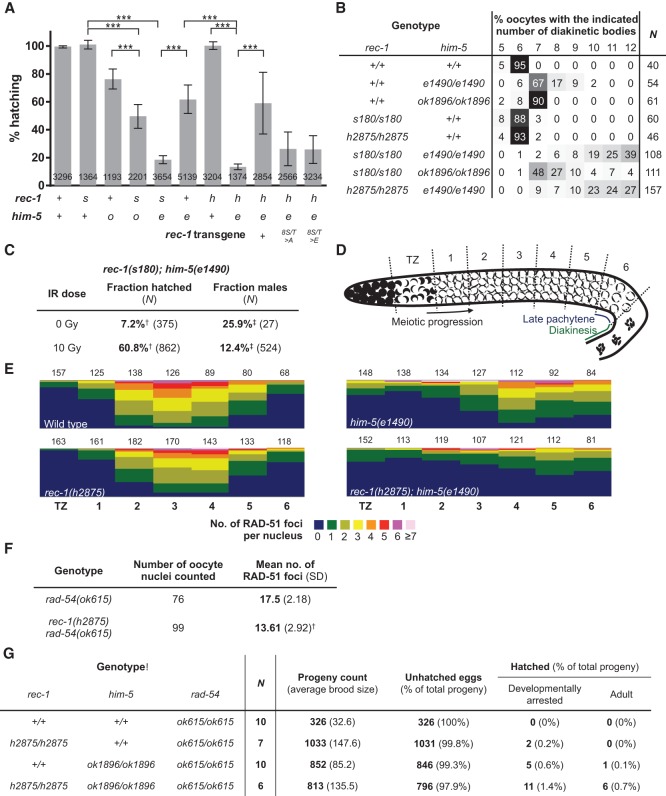
*rec-1* and *him-5* mutants interact genetically to alter the number of crossover events. (*A*) The *rec-1; him-5* double mutants exhibit lower hatching frequency than the *rec-1* and *him-5* single mutants. Eggs laid by developmentally synchronized animals were collected in 24-h intervals, and the hatched progeny were counted 3 d later. (***) *P* < 0.0001 (Kruskal-Wallis nonparametric test). Values on the bars indicate the number of eggs counted for the specific genotype. The *rec-1* genotypes used were wild type (+), *s180* (*s*), *h2875* (*h*), a phospho-mutant allele (*8S/ T> A*), and a phospho-mimetic allele (*8S/T* > *E*). The *him-5* genotypes used were wild type (+), *ok1896* (*o*), and *e1490* (*e*). (*B*) *rec-1* and *him-5* mutations act synergistically to increase the number of 4′,6-diamidino-2-phenylindole (DAPI)-staining structures in the diakinetic oocyte. For added clarity, cells in the table are shaded on a gradient—0 (white) to 100 (black)—based on value. (*N*) Number of oocytes assayed. (*C*) Consistent with a lack of meiotic crossovers, the fraction of hatched *rec-1(s180); him-5(e1490)* embryos increased, and the fraction of spontaneous *rec-1; him-5* male progeny decreased by treatment with ionizing radiation, an exogenous source of DSBs that can promote crossovers in the absence of SPO-11 function (Dernburg et al. 1998). (^†^) The difference is statistically significant (*P* ≈ 4.9 × 10^−68^, χ^2^-test); (^‡^) the difference is statistically significant (*P* ≈ 0.042, χ^2^-test). (*D*) Schematic of the *C. elegans* germline showing regions in which RAD-51 foci were quantified in individual nuclei. (*E*) Quantification of the percentage of nuclei (*Y*-axis) in each region shown in *D* (*X*-axis) with the number of RAD-51 foci (revealed as a heat map showing the range of foci from 0 to 7). Numbers *above* each stacked bar indicate the numbers of nuclei examined per region from TZ through zone 6. Two gonad arms were analyzed for each genotype. (*F*) A loss of function in *rec-1* reduces the total number of DSBs in late pachytene nuclei, as assayed by RAD-51 staining in *rad-54(ok615)* mutants, which cannot process meiotic DSBs into recombination intermediates. (^†^) The reduction compared with *rad-54(ok615)* is significant (*P* < 0.0001, two-tailed Student's *t*-test). (*G*) Mutations in *rec-1*, *him-5*, or both rescue the maternal-effect embryonic-lethal phenotype of *rad-54(ok615)* in two ways: by increasing the number of eggs laid (“progeny count”) and by increasing the proportion of eggs that hatch into either developmentally arrested larvae (“developmentally arrested”) or larvae that eventually grow into adult animals (“adult”). Both effects are consistent with the decreased number of DSBs in the *rec-1*, *him-5*, or double-mutant genetic background.

Loss of function of *him-5* results in strong crossover suppression on the X-chromosome and an altered distribution of crossovers on both the X chromosome and the autosomes (Meneely et al. 2012). The redistribution of meiotic crossover events on the autosomes is similar to that observed for *rec-1* loss of function. The crossover distribution phenotypes, together with their putative evolutionary link, prompted us to examine their genetic relationship. *him-5* loss of function reduces hatching efficiency to 60%–70% (Meneely et al. 2012), whereas *rec-1* loss of function has no impact on hatching (Rattray and Rose 1988). In contrast to the single mutants, the *rec-1; him-5* double mutant exhibited synthetic lethality and was severely reduced for hatching efficiency relative to the single mutants (Fig. 5A,C; Supplemental Fig. 4A).

To investigate the basis of the embryonic lethality in *rec-1; him-5* double mutants, we first examined diakinesis stage oocytes. At diakinesis, the homologs that have undergone an exchange event are held together as bivalents, whereas nonexchange pairs separate from one another at diplotene and condense into smaller, univalent chromosomes. Whole-mount staining with the DNA dye 4′,6-diamidino-2-phenylindole (DAPI) allows for rapid visualization of diakinetic structures. In *rec-1* mutants, almost all nuclei contain six bivalents at diakinesis, consistent with normal hatching frequencies and a very low X-nondisjunction frequency (Fig. 5B). *him-5* mutants, as previously reported (Meneely et al. 2012), contain predominantly five bivalents and two univalent (X) chromosomes, although occasionally additional univalent chromosomes are observed (Fig. 5B). In contrast, analysis of the *rec-1; him-5* double mutant revealed a significant increase (*P* < 0.0001, χ^2^-test) in the frequency of univalent chromosomes over that of single mutants (Fig. 5B). Nonexchange chromosomes segregate randomly at the first meiotic division, leading to aneuploidy in the resultant gametes. Since most aneuploidies are embryonic-lethal in *C. elegans* (Hodgkin et al. 1979), the high frequency of univalents in the *rec-1; him-5* double mutants likely explains their high incidence of embryonic lethality.

### Both *rec-1* and *him-5* facilitate the formation of meiotic DSBs

Next, we considered the possibility that the univalent phenotype of the *rec-1; him-5* double mutant reflects a defect in the induction of meiotic DSBs. If this were the case, we reasoned that the phenotype could be rescued by artificially introducing meiotic DSBs with ionizing radiation, as has been previously shown for *spo-11* and *him-5* mutants (Dernburg et al. 1998; Meneely et al. 2012). As shown in Figure 5C, ionizing radiation substantially suppressed the embryonic lethality of *rec-1; him-5* double mutants, suggesting that defects in meiotic DSB formation are the major underlying cause of the observed synthetic lethality. To rule out that pairing and/or synapsis defects contribute to the crossover deficit, we used immunohistochemistry to monitor these processes. In *rec-1* and *him-5* single mutants as well as the *rec-1; him-5* double-mutant animals, pairing and synapsis were indistinguishable from wild type (Supplemental Fig. 5). Together, these data reveal that loss of *rec-1* function enhances the meiotic DSB formation defect of *him-5*, leading to a significant increase in nonexchange chromosomes and ensuing embryonic lethality.

The exacerbation of the *him-*5 DSB defect by *rec-1* raised the possibility that *rec-1* itself may be inefficient in meiotic DSB formation. Using immunostaining for the DNA strand exchange protein RAD-51 as a marker for meiotic DSBs, we observed differences in both the number and temporal localization of RAD-51 in *rec-1* single mutants and *rec-1; him-5* double mutants. In wild type, we counted an overall average of just under three RAD-51 foci per oocyte nucleus, with the most abundant signal in the midpachytene stage of meiosis, which corresponds to zone 3 in Figure 5, D and E, where some nuclei with six to seven RAD-51 foci can be seen. In *rec-1* and *him-5* single mutants, there were fewer RAD-51 foci overall (an average of one per nucleus in the total count), and in *him-5* mutants, a notable shift in the presence of foci at the later stages of prophase can be seen (Fig. 5E; Meneely et al. 2012). This phenotype was exacerbated in the *rec-1; him-5* double mutant; an overall average of 0.5 foci per nucleus was detected, and the majority of these were in the later stages—zones 4, 5, and 6—with the addition of some larger, brighter foci that are possible indicators of repair defects (Fig. 5D,E; Supplemental Fig. 6; Z Kessler, N Macaisne, and JL Yanowitz, unpubl.). Because *rec-1* mutants do not exhibit embryonic lethality or an increase in male progeny, the reduction in the number of DSBs must be small. To quantify the total number of DSBs generated in *rec-1*, we quantified RAD-51 foci in late pachytene (Fig. 5D, zone 6) in a *rad-54(ok615)* mutant background that accumulates single-stranded RAD-51 filaments that cannot be further processed into exchange intermediates (Mets and Meyer 2009). In these experiments, we observed an average of 17.5 RAD-51 foci in *rad-54(ok615)* single mutants but only 13.6 foci in *rec-1(h2875); rad-54(ok615)* double mutants (*P* < 0.0001, two-tailed *t-*test) (Fig. 5F). These data support the conclusion that there is a mild defect in DSB formation in the *rec-1* mutant. Furthermore, the observation that *rec-1; him-5* double mutants exhibit significantly reduced numbers of meiotic RAD-51 foci compared with either single mutant supports the conclusion that HIM-5 and REC-1 act redundantly during the generation of meiotic DSBs.

To provide further evidence in support of a role for *rec-1* and *him-5* in meiotic DSB formation, we examined genetic interactions with *rad-54* mutants. RAD-54 is essential for meiotic DSB repair by homologous recombination; in its absence, meiotic DSBs are aberrantly repaired by nonhomologous end-joining, which manifests as chromatin aggregates at diakinesis (Ward et al. 2010). *rad-54* mutations severely impact fertility, producing very few eggs (an average of 32) (Fig. 5G) due to massive apoptosis of germline nuclei (Stergiou et al. 2011), and all of the eggs that are laid fail to hatch (Fig. 5G). Mutating *rec-1*, *him-5*, or both *rec-1* and *him-5* increased the number of eggs laid and the number of hatched progeny in the *rad-54* mutant background (Fig. 5G). The partial rescue of the *rad-54* mutant phenotype by *rec-1; him-5* is similar to that seen with mutants defective for meiotic DSB formation, including *spo-11* (Stergiou et al. 2011), which reinforces a role for *rec-1* and *him-5* at this stage of meiosis I.

## Discussion

In all of the species examined, the numbers of meiotic crossovers per unit DNA differ along the chromosome. This is seen dramatically in *C. elegans* due to near complete crossover interference along the autosomes, resulting in a single crossover per homolog pair and producing a recombination map with apparent gene clusters in the central region (Brenner 1974; Barnes et al. 1995). The apparent clustering of crossovers is eliminated by mutation in the *rec-1* gene (Rose and Baillie 1979; Zetka and Rose 1995), but the identity of the mutation responsible for this phenotype remained a mystery for >30 years. In this study, we report the molecular identification of the *rec-1* gene, which was the first genetic locus described that compromises the normal distribution of meiotic crossovers along the chromosome in any organism. We present evidence that the REC-1 protein contains a repeated motif, which is a CDK substrate in vitro, and its phosphorylation and subsequent dephosphorylation appear to be required for establishing the normal distribution of meiotic crossover in vivo. Our phylogenetic analysis also revealed that REC-1 is evolutionarily related to HIM-5, albeit distantly, yet functionally, REC-1 and HIM-5 cooperate to promote efficient meiotic break formation.

In species where genomic and RNA sequences are available, only those species within the *elegans–japonica* clade have a gene in the same relative orientation as *C. elegans rec-1* and situated between the *pif-1* and *y18h1a.3* orthologs. Intriguingly, with the exception of *rec-1*, these positionally equivalent genes encode proteins that all share sequence similarity centered on an R-F-x-x-L-P/S box situated at the N terminus. Using the sequence similarity shared by these R-F-x-x-L-P/S-box genes to search the *C. elegans* genome, we identified *him-5* as the most significant hit. One reasonable possibility is that, after the divergence of *C. elegans* from other *Caenorhabditis* spp., the ancestral *rec-1/him-5* gene duplicated and became the present-day *rec-1* and *him-5.* In other species in the *elegans–japonica* clade, there remains only a single R-F-x-x-L-P/S-box gene within this synteny block. Examples of poor sequence conservation can also be found in genes encoding Spo11 accessory factors in closely related yeasts (Richard et al. 2005; Keeney 2008). It has been proposed that the divergence of meiotic genes inhibits the reproductive success of interspecific hybrids (Swanson and Vacquier 2002). Thus, the divergence of the REC-1/HIM-5 orthologs may be a reflection of this principle.

Divergent as *rec-1/him-5* orthologs may be, the preference for recombination in chromosomal arms is, intriguingly, preserved through evolutionary time. Emerging genetic and genomic analyses in other *Caenorhabditis* species indicate that recombination events, as in *C. elegans*, preferentially take place on the chromosome arms (Ross et al. 2011; M. Rockman, pers. comm.). Clearly, the selective pressure for this preference exists among the *Caenorhabditis* species, but, given the poor sequence conservation of *rec-1/him-5* orthologs, the components responsible for this preference may be evolving rapidly.

The observation of a genetic interaction between *rec-1* and *him-5* clarified the relationship between these two genes, which were known to have similar mutant phenotypes, at least in relation to their impact on crossover distribution. The significant reduction in the number of meiotic RAD-51 foci in the *rec-1; him-5* double mutant shown here also implicates *rec-1* and *him-5* in meiotic DSB formation and suggests that the latter is a key factor in determining crossover distribution. Furthermore, our observation that wild-type *rec-1*, but not the phospho-mutant *dwSi6[rec-1(8S/T* > *A)]* or the phospho-mimetic transgene, rescues the redistribution of crossovers in the *rec-1* mutant as well as the synthetic lethality of the *rec-1; him-5* double mutant reveals that these processes are controlled in part by the phosphorylation status of REC-1. While the kinase responsible for REC-1 phosphorylation in vivo remains to be clearly defined, our data suggest that this kinase is likely to be a member of the CDK family. This is suggested by our observations that (1) REC-1 contains consensus CDK phosphorylation sites (S/T-P) within each of the four repeats, (2) these S/T-P sites are phosphorylated by recombinant CDK in vitro, and (3) REC-1 is phosphorylated on these S/T-P sites in *C. elegans* extracts, and this is abolished by roscovitine, a specific CDKi, and not by inhibitors of other kinases.

Our data revealed that *rec-1* and *him-5* function cooperatively to ensure the induction of a wild-type level of DSBs. Although there are significant differences between the process of meiosis in yeast and worms and between their respective meiotic mutant phenotypes, we speculate that REC-1 and HIM-5 may function as SPO-11 accessory proteins analogous to those described in *Saccharomyces cerevisiae* (for review, see Keeney 2001, 2008; de Massy 2013). In *S. cerevisiae*, meiotic DSB formation is dependent on the phosphorylation of Mer2 by CDK and Dbf4-dependent kinase (DDK) (Henderson et al. 2006; Wan et al. 2008; Murakami and Keeney 2014). Thus, a functional parallel can be drawn between the two CDK-dependent mechanisms that determine the position of meiotic crossover events.

In summary, loss of function of the *rec-1* gene eliminates the wild-type preference for where a crossover will occur in *C. elegans* without severe accompanying phenotypic consequences. The molecular identification of the gene product responsible for this phenotype has not only provided information about how the crossover pattern is determined in this species but placed *rec-1* among a category of genes with divergent sequence that is required for crossover placement, and, in conjunction with HIM-5, REC-1 is required for efficient meiotic DSB formation.

## Materials and methods

### Worm strains used

Unless otherwise noted, strains were kept at 20°C on NGM agar seeded with *Escherichia coli* strain OP50 as previously described (Brenner 1974). BC313 *rec-1(s180) (I)* was isolated as described (Rose and Baillie 1979). Additional mutations in *rec-1* were generated by directed mutagenesis using CRISPR–Cas9 protocols described previously (Friedland et al. 2013). Strains with transgenic *rec-1* alleles were generated using *Mos*SCI (Frøkjær-Jensen et al. 2008) by microinjection into Unc-119 segregants from strain EG6699 [*ttTi5605 (II)*; *unc-119(ed3) (III)*; *oxEx1578*]. This strain was obtained from the *Caenorhabditis* Genetics Center (CGC), funded by the National Institutes of Health Office of Research Infrastructure Programs (P40 OD010440). The two *him-5* mutant isolates used in our study (*e1490* and *ok1896*) were characterized previously (Meneely et al. 2012) and are archived at the CGC. Additional strains and their genotypes are listed in Supplemental Table 1.

### Reagents for microinjection

The plasmids containing *cas9* and the synthetic sgRNA gene originated from the Calarco laboratory (Friedland et al. 2013) and were requested from Addgene (Addgene IDs 46168 and 46169, respectively). The remaining reagents and the overall protocol were largely based on the work of Friedland et al. (2013) with minor modifications as detailed in the Supplemental Material.

Integrated *rec-1* transgenic lines were made as described previously by injection into Unc-119 segregants from EG6699 [*ttTi5605 (II); unc-119(ed3) (III); oxEx1578*] (Frøkjær-Jensen et al. 2008). Integrated wild-type *rec-1(+)*, phospho-mutant *rec-1(8S/T* > *A)*, and phospho-mimetic *rec-1(8S/T* > *E)* were designated *dwSi4[rec-1(+) Cbr-unc-119(+)]*, *dwSi6[rec-1(8S/T* > *A) Cbr-unc-119(+)]*, and *dwSi5[rec-1(8S/T* > *E) Cbr-unc-119(+)]*, respectively.

### Determination of recombination frequency

The scoring of recombination events using visible markers was adapted from Zetka and Rose (1995). The protocol for the determination of oocyte-specific crossover events using snip-SNPs was adapted from Mets and Meyer (2009) with modifications to optimize the PCR and restriction reactions.

### In vitro expression and phosphorylation of REC-1

Wild-type REC-1 and phospho-mutant REC-1(8S/T > A) fused to glutathione-S-transferase (GST) at the N terminus and separated by a PreScission protease site were expressed in Sf9 cells using the baculovirus expression system (Invitrogen). One liter of Sf9 cells was infected (multiplicity of infection [MOI] = 5) at a concentration of 10^6^ cells per milliliter and harvested 72 h after infection. The cell pellet was lysed in a lysis buffer (50 mM Tris-HCl at pH 8.0, 0.5 M NaCl, 0.1% NP-40, 1 mM EDTA, 1 mM DTT, 0.2 mM PMSF, 2× Complete protease inhibitor [Roche]) and sonicated three times on ice (30 sec at maximum amplitude with 2-min rest intervals). After a 30,000*g* centrifugation for 30 min at 4°C, the GST-tagged proteins in the lysate supernatant were immobilized on glutathione-Sepharose 4B (GE Healthcare) and washed several times with a kinase buffer (50 mM Tris-HCl at pH 7.5, 1 mM DTT, 100 µM sodium orthovanadate, 5 mM MgCl_2_, 0.1 µM ATP) in preparation for phosphorylation analysis.

In vitro phosphorylation was performed on 25 μL of each immobilized recombinant REC-1 protein substrate [GST::REC-1(+) and GST::REC-1(8S/T > A)] mixed with 100 ng of CDK4/cyclin D3 kinase per reaction (gift from Tohru Takaki) and 1 µCi of [^32^P] γ-ATP in a 50-μL reaction inside micro bio-spin chromatography columns (Bio-Rad). After a 10-min incubation at 20°C in the kinase buffer, the reaction was terminated by the addition of a stop buffer (50 µM ATP, 5 mM EDTA, 0.1% [v/v] Triton X-100). Unreacted radioactive ATP was separated from the substrate by multiple washes with an elution buffer (50 mM Tris-HCl at pH 7.0, 150 mM NaCl, 0.5 mM EDTA, 1 mM DTT, 0.01% Triton X-100). The wild-type and mutant GST::REC-1 substrates were then cleaved from the Sepharose matrix by incubation with PreScission protease (GE Healthcare) for 1 h at 20°C. The phosphorylation product was eluted, separated by SDS-PAGE gel electrophoresis, and visualized by autoradiography using a Typhoon PhosphorImager (GE Healthcare).

### Peptide arrays and kinase assays

For the peptide array studies, 350 fragments of 20-mer peptides juxtaposed by one amino acid scanning the complete REC-1 protein were synthesized and spotted onto cellulose membrane. The membrane was activated by soaking in methanol for 2 min and washed twice with kinase buffer supplemented with 3% BSA. In vitro phosphorylation was performed by incubating the membrane in 1 mL of kinase buffer supplemented with 50 μg of CDK4/cyclinD3 or N2 worm extract (protein concentration of 10 mg/mL) and 100 µCi of [^32^P] γ-ATP. Extracts were supplemented with 5 µM CDKi/roscovitine (Sigma) for 1 h at 37°C. After adding stop buffer, the membrane was washed sequentially in 1 M NaCl, then 1% SDS, and finally 0.5% phosphoric acid solution. After washing in 96% ethanol, the membrane was dried and exposed to autoradiography film. An identical array stained with Ponceau (Sigma) was used as a control to visualize the presence of peptide spots. For kinase assays on peptides, we used 15 μg of the following peptide: Bio-SSPSPKSPRFAEKPSEIAKS (S = Ser146). Kinase assays were performed in 30 μL of kinase buffer (50 mM TrisHCl at pH 7.5, 10 mM MgCl_2_, 5 mM DTT) with 10 µg/mL *C. elegans* N2 extracts and 100 µCi of γ-^32^P-ATP for 1 h at 37°C. Reactions were supplemented with 5 µM kinase inhibitors caffeine (Sigma), ATMi/KU-55933 (Abcam), ATRi/VE-821 (Selleckchem), CHK1i/UCN01 (Sigma), CDKi/roscovitine (Sigma), and PLK1i/BI2536 (Selleckchem). The reaction was stopped by the addition of 5 μL of 0.5M EDTA (pH 8.0), and the reactions were spotted onto 2.1-cm-diameter Whatman P81 cellulose phosphate filter circles. The circles were washed three times in cold 0.5% phosphoric acid and once with acetone, dried at room temperature, and put into scintillation vials with 5 mL of scintillation liquid (Ecoscint A, National Diagnostics), and the scintillation was measured.

### Identification and analysis of *rec-1* and *him-5* orthologs in several *Caenorhabditis* species

Based on previously published genomes, their respective gene annotations, and the RNA sequencing data deposited at WormBase (WS243) and ModENCODE (Celniker et al. 2009), we identified the synteny block containing *rec-1* in six other *Caenorhabditis* species. Using the putative ORFs around *rec-1* and the relative positions and orientations of their orthologs in the six *Caenorhabditis* species, we identified a putative ORF that is positionally equivalent to *rec-1*. Subsequent profile-based similarity searches employed HMMer (Finn et al. 2011) against the UniRef50 database (Suzek et al. 2007) using an alignment of the N-terminal region conserved in the *Caenorhabditis* proteins (including *C. briggsae*, *C. sinica* [sp. 5], *C. brenneri*, *C. remanei*, *C. tropicalis*, and *C. japonica*) encoded by genes syntenic with *C. elegans rec-1*. These searches identified the *C. elegans* HIM-5 protein sequence as being statistically significantly similar to these proteins (*E* = 6 × 10^−3^).

### Immunofluorescence and microscopy

Fixation and immunostaining of gonads were performed as described (Chan et al. 2003). The following antibodies were used (at the specified concentrations): rabbit anti-RAD-51 (1:1000) (Rinaldo et al. 2002), guinea pig anti-HIM-8 (1:500) (Phillips et al. 2005), and anti-SYP-1 (1:2000) (Colaiácovo et al. 2003). Corresponding secondary antibodies conjugated to Alexa 488, Alexa 568, and Alexa 633 were obtained from Invitrogen and used at 1:1000 to 1:2000 dilution. Immunostained tissues were then mounted in Prolong Gold with DAPI (Invitrogen) and imaged on a Nikon A1r confocal microscope (Nikon Instruments) in 0.2-μm increments on the *Z*-axis. Analysis of stained nuclei was carried out as described (Colaiácovo et al. 2003).

## Supplementary Material

Supplemental Material
