# Supplementary material for: REC-1 and HIM-5 distribute meiotic crossovers and function redundantly in meiotic double-strand break formation in Caenorhabditis elegans
Source: Genes Dev. 2015 Sep 15;29(18):1969–79. doi: 10.1101/gad.266056.115 (PMC4579353; doi:10.1101/gad.266056.115)
Supplement: Supplemental Material [file supp_29_18_1969__index.html]

Supplemental Material 

# REC-1 and HIM-5 distribute meiotic crossovers and function redundantly in meiotic double-strand break formation in *Caenorhabditis elegans*

## Supplemental Material

**Files in this Data Supplement:**

- Supplemental Figures.pdf
- Supp Material.docx
