# Supplementary material for: REC-1 and HIM-5 distribute meiotic crossovers and function redundantly in meiotic double-strand break formation in Caenorhabditis elegans
Source: Genes Dev. 2015 Sep 15;29(18):1969–79. doi: 10.1101/gad.266056.115 (PMC4579353; doi:10.1101/gad.266056.115)

Supplemental Figures

Supplemental Figure 1

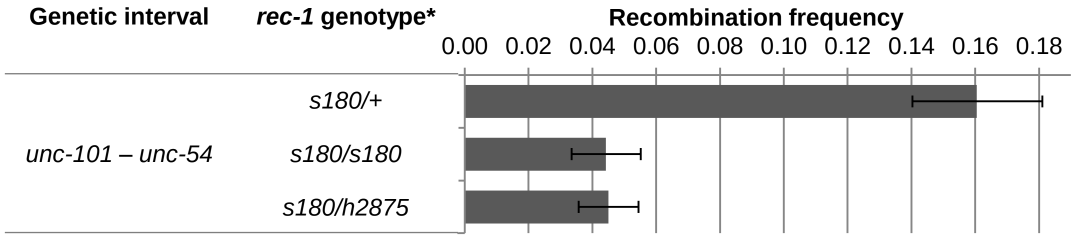

Supplemental Figure 2

A

Gamete type	Genotype at Site A*	Site B	Site C	Site D	Site E	Numbers observed in *rec-1(+)*	*rec-1(h2875)*
i	*B*	*B*	*B*	*B*	*B*	38	27
ii	*H*	*H*	*H*	*H*	*H*	29	41
iii	*B*	*H*	*H*	*H*	*H*	8	5
iv	*H*	*B*	*B*	*B*	*B*	10	2
v	*B*	*B*	*H*	*H*	*H*	1	6
vi	*H*	*H*	*B*	*B*	*B*	2	5
vii	*B*	*B*	*B*	*H*	*H*	2	4
viii	*H*	*H*	*H*	*B*	*B*	1	7
ix	*B*	*B*	*B*	*B*	*H*	4	4
x	*H*	*H*	*H*	*H*	*B*	3	5
with >1 crossover	(not observed)					0	0
					Total	98	106

B

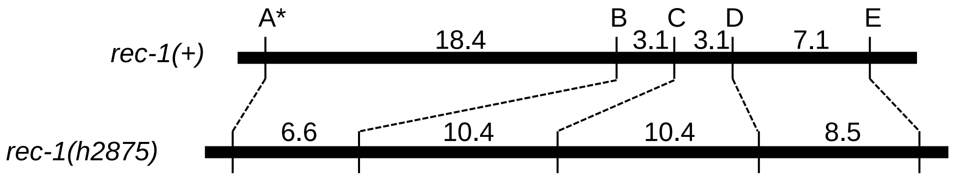

Supplemental Figures

Supplemental Figure 3

```
CLUSTAL 2.1 multiple sequence alignment


Celeg_rec-1-N      MPGNDDIVMLSDEIVADLRRFIASRPVIQFDRRPGYVPTPWRNCWKEIIEIWSDDDSEDD 60
Crem_rec-1-N       MEDDDDLIEITDEQHARIRATIGKRP--EFDH---LILRDLQLSDSEEHRCESEEESEDD 55
                   * .:**:: ::**  * :*  *..**  :**:    :    : . .*   . *:::****

Celeg_rec-1-N      DGD----------VITGSQEAQLKR 75
Crem_rec-1-N       EGGELFYKRNTLRVTTVAQEAAFKR 80
                   :*.          * * :*** :**
```

Supplemental Figure 4

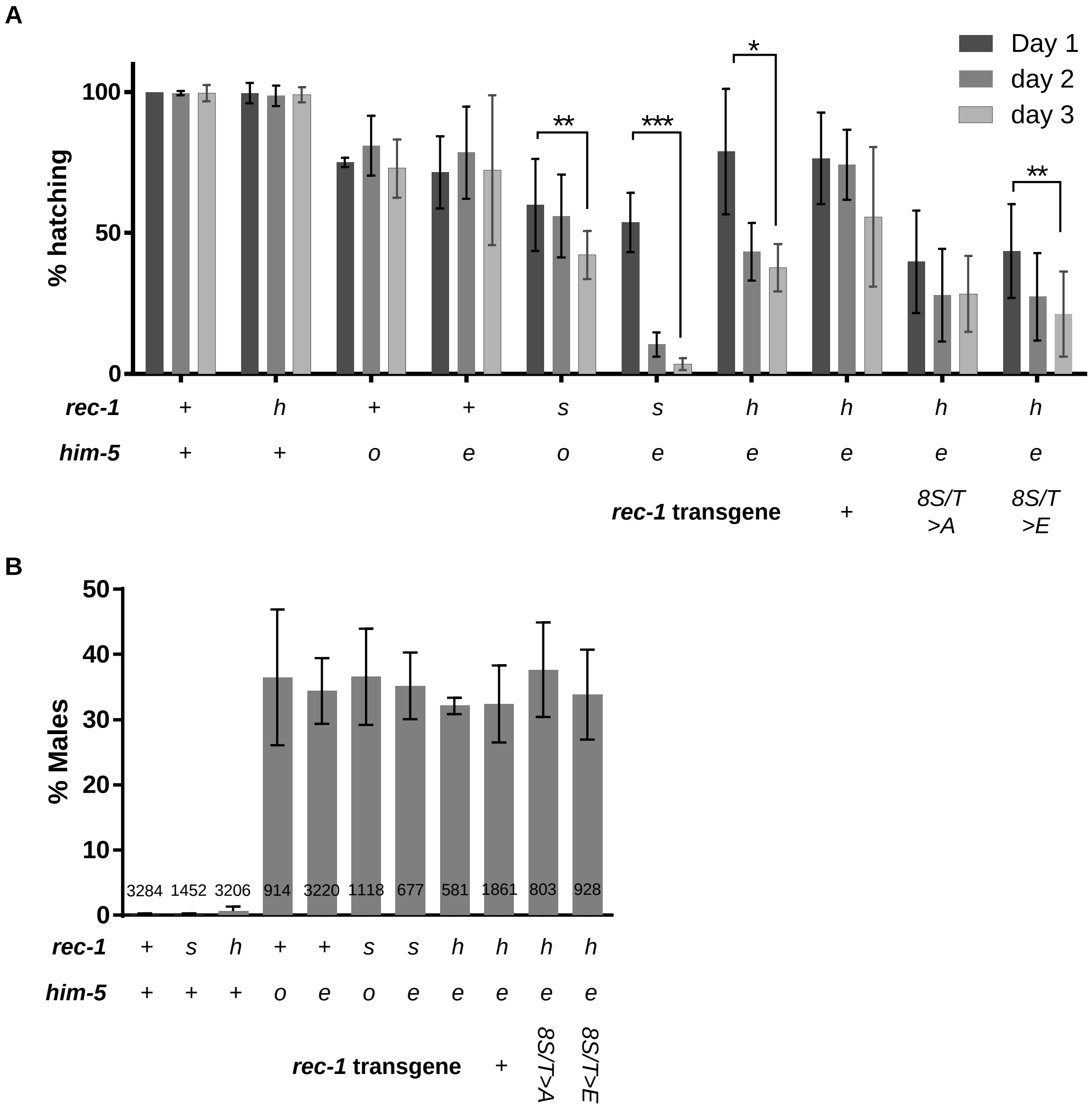

Supplemental Figure 5

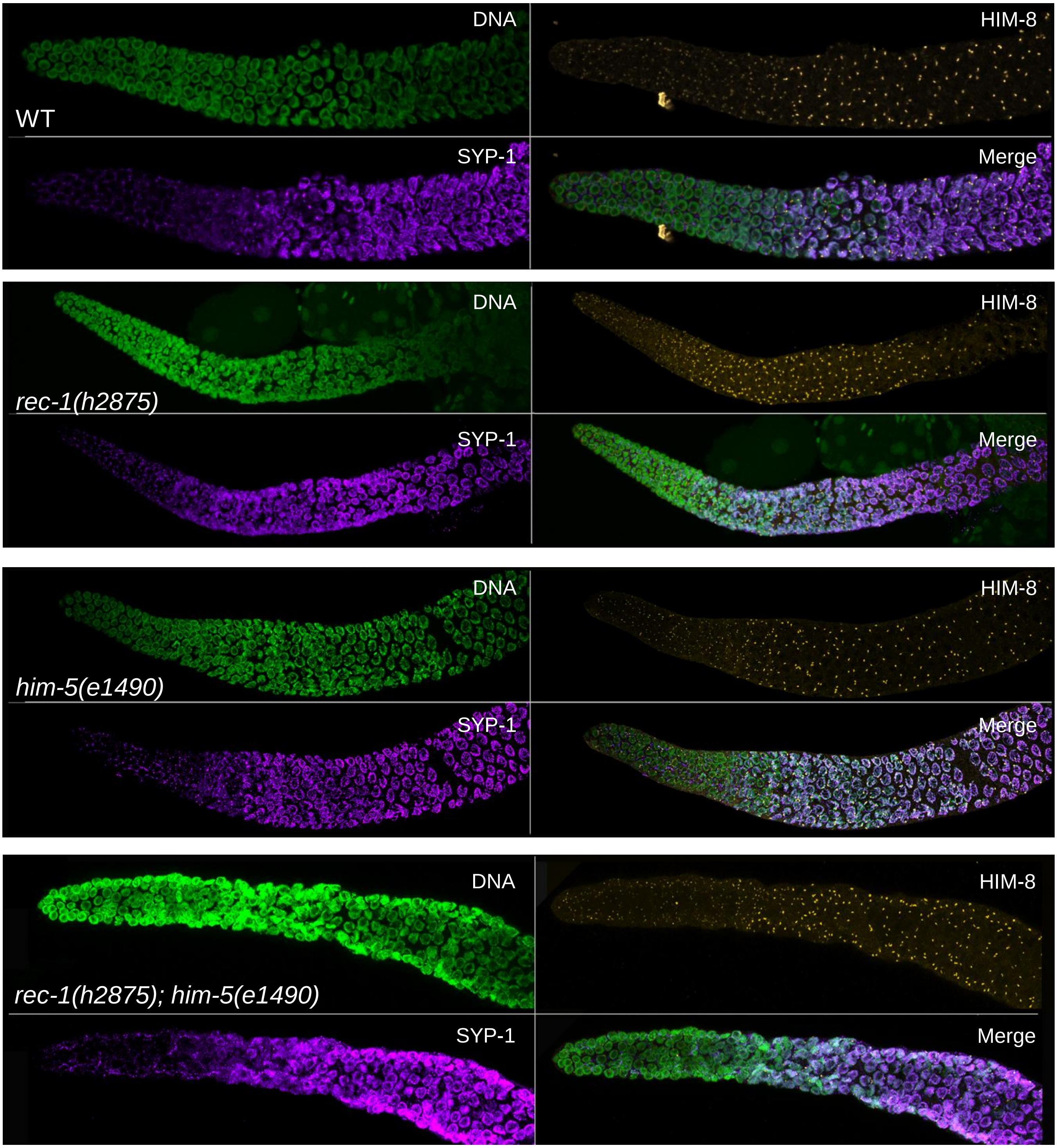

Supplemental Figure 6

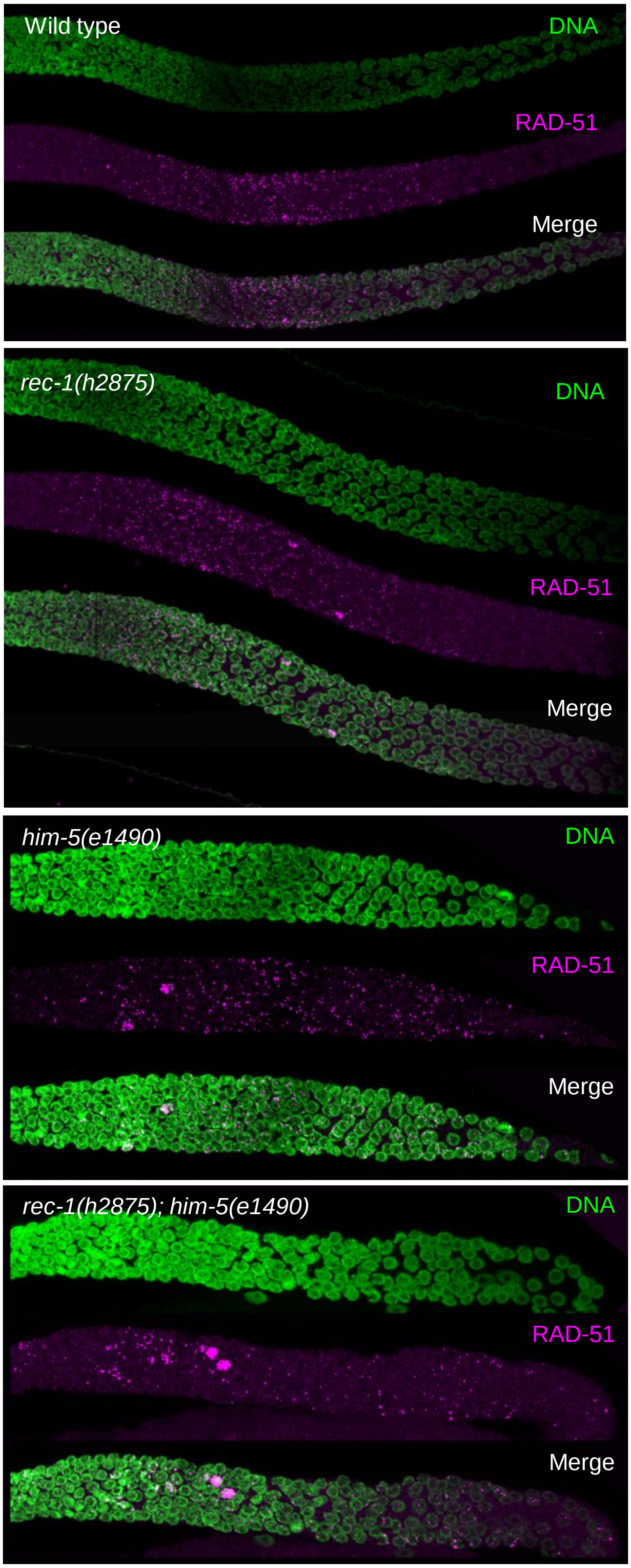

Supplement: Supplemental Material [file supp_29.18.1969_Supplemental_Figures.pdf]
