# Supplementary material for: REC-1 and HIM-5 distribute meiotic crossovers and function redundantly in meiotic double-strand break formation in Caenorhabditis elegans
Source: Genes Dev. 2015 Sep 15;29(18):1969–79. doi: 10.1101/gad.266056.115 (PMC4579353; doi:10.1101/gad.266056.115)
Supplement: Supplemental Material [file supp_29.18.1969_SuppMaterial.docx]

# Supplemental Material for:

REC-1 and HIM-5 distribute meiotic crossovers and function redundantly in meiotic double-strand break formation in *Caenorhabditis elegans*

George Chung^1^, Ann M. Rose^1^, Mark I.R. Petalcorin^2^, Julie S. Martin^2^, Zebulin Kessler^3^, Luis Sanchez-Pulido^4^, Chris P. Ponting^4^, Judith Yanowitz^3^* and Simon J. Boulton^2^*

^1^Department of Medical Genetics, University of British Columbia, Vancouver, Canada
^2^DNA Damage Response Laboratory, London Research Institute, Clare Hall Laboratories, South Mimms, EN3 3LD, UK.
^3^Magee-Womens Research Institute, University of Pittsburgh School of Medicine, Pittsburgh, PA 15213
^4^MRC Functional Genomics Unit, Department of Physiology, Anatomy and Genetics, University of Oxford

**This section includes:**

Supplemental Materials and Methods

Author contributions

References for Supplemental Materials

Supplemental Tables 1 and 2

Supplemental Figs. 1 to 5

## Supplemental Materials and Methods

### *Reagents for generating* rec-1 *mutant and transgenic alleles*

Plasmid originated from the Calarco lab (Friedland et al. 2013) were requested from Addgene (Addgene ID 46168 and 46169, respectively). The remaining reagents and the overall protocol were largely based on the work of Friedland *et al.* (Friedland et al. 2013) with the following modifications. The sgRNA target sequence was changed to 5'-GAACTGGATAACTGGCCGGC-3', found on the anti-sense strand in the second exon of *rec-1.* Co-injection markers, *myo-2::GFP* (GFP expressed in pharyngeal muscle) and *myo-3::GFP* (body wall muscle), came from the Fire lab and used in lieu of the corresponding mCherry markers used by Friedland *et al*. The microinjection mixture concentrations were altered slightly: 45 ng/μL of the plasmid containing *cas9*, 45 ng/μL of the plasmid containing the synthetic sgRNA gene, 2.5 ng/μL of the plasmid containing *Pmyo-2::GFP*, and 5 ng/μL of the plasmid containing *Pmyo-2::GFP*. This was microinjected into one or both gonad arms of young adult hermaphrodite animals (Kadandale et al. 2009). It was hoped that a palindromic oligo that would aid in genotyping would integrate at the site of the Cas9 incision (but it never did), and this oligo was added to the micro-injection mix at 5 ng/μL. This oligo was not detected by genotyping experiments and appeared to have been inconsequential to the outcome of the experiments.

Targeting transgenes containing wild type *rec-1*, phospho-mutant *rec-1(8S/T>A)*, and phospho-mimetic *rec-1 (8S/T>E)* were constructed by synthesizing DNA fragments (GeneArt) and cloning them to pCFJ151 using standard techniques (Frøkjær-Jensen et al. 2008). All cloning PCR amplifications were done with Q5 high-fidelity DNA polymerase (New England Biolabs). Integrated *rec-1* transgenic lines were made as described previously by injection into Unc-119 segregants from EG6699 - *ttTi5605 (II); unc-119(ed3) (III); oxEx1578* (Frøkjær-Jensen et al. 2008).

### *Finding a suitable target for Cas9 cleavage*

The goal of our targeted mutagenesis was to generate a null allele of *y18h1a.7* that would phenocopy the existing *s180* allele of *rec-1*. Several sites at the 5’ end of *y18h1a.7* could potentially lead to deletions of the start codon; however, this was not ideal as there was a second methionine residue not too far downstream, which could be used as an alternative translation start site, thus generating a nearly wild type protein. This favoured a choice of a Cas9 target site in the following exon (exon 2). We noted that a C→T non-sense mutation within the second exon of *y18h1a.7* was a candidate for the *s180* mutation(Rose et al. 2010), so an out-of-frame deletion in this exon would likely phenocopy the effects of *rec-1(s180)*. Thus, a Cas9 target site was chosen, overlapping a *Nae*I restriction site which could be used for genotyping this locus (Fig. 1). This Cas9 target site was cloned into the plasmid containing the synthetic sgRNA gene, replacing the original target sequence derived from *unc-119*.

### *Screening for y18h1a.7 mutants*

Mutations in *y18h1a.7,* a candidate for *rec-1*, were not expected to have a morphological or developmental phenotype*.* All mutant screening, therefore, was done by Sanger sequencing at the *y18h1a.7* locus.

GFP-expressing hermaphrodite F_1_ animals arising from the injected P_0_ were individually plated, allowed to lay eggs for one day, then treated with lysis buffer (30 mM Tris pH 8,100 mM NaCl, 0.7% Tween 20 and 100 μg/ml proteinase K) at 57°C for 1 hour to extract the DNA and then at 95°C for 15 minutes to inactivate the proteinase K. The 5’ end of the *y18h1a.7* locus was then amplified by PCR using primers “y18 CRISPR seq Fi” (CGCCATGTGCCTTTAGTACC) and “y18 CRISPR seq Ri” (GCAGTTTCTGGTGATTTTCG) with the program “CO_55” (94°C for 4:00; then 34 rounds of 94°C for 0:30, 55 °C for 0:30, and 72°C for 1:00; ending with 72°C for 10:00). The expected wild type band size was 465 bps. Sanger sequencing was then performed with one of the two primers used for PCR. The F_1_ animals with obvious double peaks in their Sanger chromatograms were likely heterozygous for a mutation in *y18h1a.7*. Their progeny were kept and the mutation was made homozygous over an additional 1 to 2 generations.

### *Determination of the recombination frequency in a defined genetic interval with mutations causing visible phenotypes*

The *rec-1(s180)* homozygous animals were known to affect the distribution of crossover events across the chromosome, but the overall frequency of meiotic crossovers across individual chromosomes remained roughly 0.5(Zetka and Rose 1995). This meant that recombination frequency increases at certain intervals were compensated by decreases in other intervals. Two well characterized genetic intervals were chosen for this work: *dpy-5 – unc-13*, where the recombination frequency increased in *rec-1(s180)* homozygotes compared to wild type animals, and *unc-101 – unc-54*, where the recombination frequency decreased in *rec-1(s180)* homozygotes compared to wild type animals(Zetka and Rose 1995).

Hermaphrodite animals, with or without *rec-1* mutations and homozygous for *dpy-5(e61)* and *unc-13(e51),* were mated with homozygous *rec-1(+)* or *rec-1(-)* males (the ‘-’ allele denotes either *s180*, *h2875* or *h2872*). The non-Dpy, non-Unc F_1_ hermaphrodites, carrying *dpy-5(e61) unc-13(e51)* in *cis,* were allowed to self. All of their progeny in the F_2_ were scored based on the phenotypes: wild type, Dpy, Unc or DpyUnc. We then calculated the recombination frequency in this interval using the formula $1-\sqrt{1-2R}$, where $R=\frac{\left[ Number of Dpys+number of Uncs \right]}{\left[ Total number of progeny \right]}$ (Brenner 1974; Zetka and Rose 1995). This recombination frequency calculation takes into account recombination events in both the egg and the sperm from the hermaphrodite.

Initially, to avoid ascertainment bias (*i.e.* the experimenter looking specifically for Dpy or Uncs on a plate arising from an F_1_ *h2875* homozygote), we scored all the F_2_ animals blindly, with their *rec-1* genotype kept a secret and only revealed to the experimenter after all the F_2_ animals were counted. The single-blind scoring was repeated by another member in the lab.

For the *unc-101 – unc-54* interval, hermaphrodite animals bearing heterozygous mutations *unc-101(m1)* and *unc-54(e190)* in *cis* were allowed to self, with or without *rec-1(-)*, and all their progeny were scored based on the movement phenotypes: wild type, Unc-101 and Unc-54. Because the movement phenotypes of *unc-54(e190)* single mutants and *unc-101(m1) unc-54(e190)* double mutants were identical, the recombination frequency was calculated using the formula$1-\sqrt{1-2R}$, where the modified$R$ is inferred to be $\frac{2\left[ number of Unc-101s \right]}{\left[ Total number of progeny \right]}$ (Brenner 1974; Zetka and Rose 1995). This recombination frequency takes into account recombination events in both the egg and the sperm from the hermaphrodite.

### *Mapping crossover events using single-nucleotide differences between N2 (Bristol) and CB4856 (Hawaii) strains of* C. elegans

The *rec-1(h2875)* was generated in an N2 (Bristol) genetic background. This original strain was outcrossed six times to strain CB4856, a Hawaiian *C. elegans* isolate that contains a set of well-characterized single-nucleotide differences from N2(Wicks et al. 2001). Separately, the original *rec-1(h2875)*-bearing strain was backcrossed six times to N2. The N2-backcrossed strain bearing *h2875* was designated KR5305; the CB4856-outcrossed strain was KR5306.

Hermaphrodite animals from KR5306 (Hawaiian) were mated with males from KR5305 (Bristol). The mated hermaphrodites were transferred to individual plates, where the appearance of F_1_ males indicated a successful cross. Outcross hermaphrodite F_1_ progeny animals were identified by the virtue of being younger than the oldest outcross male F_1_ progeny. These outcross hermaphrodite F_1_ animals were picked out as L4s (to ensure they did not mate with their siblings) and backcrossed to an N2 male. As a control, analogous crosses were performed, but starting with N2 and CB4856 instead (*i.e.* no mutation in *rec-1*). The resulting F_2_ male animals were collected for genotyping across five polymorphic sites. Each of these F_2_ animals each represented a single product of an F_1_ oocyte meiosis.

The collected F_2_ males were treated with lysis buffer (30 mM Tris pH 8,100 mM NaCl, 0.7% Tween 20 and 100 μg/ml proteinase K) at 57°C for 1 hour to extract the genomic DNA and then at 95°C for 15 minutes to inactivate the proteinase K. The genomic DNA was divided into 5 aliquots for 5 PCR reactions using the primer pairs (listed in Supplementary Table 2 with the program “CO_55” (94°C for 4:00; then 34 rounds of 94°C for 0:30, 55 °C for 0:30, and 72°C for 1:00; ending with 72°C for 10:00). The PCR products were digested, without purification, with *Aat*II, *Spe*I, *Acc*I, *Dra*I and *Hind*III (New England Biolabs) according to the manufacturer’s specifications. The five sites were chosen based on the work by Mets and Meyer (2009) with modifications to optimize the PCR and restriction reactions(Mets and Meyer 2009). From the five genotypes across the chromosome, it was possible to deduce where a recombination event took place in the F_1_ and if there were multiple crossover events in the hermaphrodite oocyte.

### *Brood analysis and irradiation*

Healthy hermaphrodites grown at 20°C were individually plated at the L4 stage. The animals were transferred to new plates once every 24 hours until the egg-laying stopped. Eggs laid were immediately counted. When each brood reached adulthood, the total number of live animals per brood was counted and checked against the egg count to give the total brood size and an estimate of the embryonic lethality frequency. The number of male progeny animals was also noted.

For brood analysis after irradiation, 24-hour post-L4 animals were exposed to 10 Gy of γ-ray from Nordion Gammacell 1000 (Ottawa ON, Canada). Eggs laid between 24 to 30 hrs post-irradiation were collected, and hatched adult animals were counted 3 days after egg-laying.

### *Analysis of tandem repeats in the* rec-1 *(DNA) and REC-1 (amino acid) sequence*

The tandem repeat structure in the third exon of *rec-1* was initially detected by visual inspection. The repeats boundaries were determined manually by maximizing the number of repeats and their sequence similarities. The corresponding repeat in the amino acid sequence was checked using the program RADAR (Heger and Holm 2000).

# Author contributions

GC, AMR, CPP, JY and SJB designed the experiments; GC, AMR, MIRP, JSM, ZK, LS-P, CPP, SJB and JY performed the experiments and analysed the data; GC, AMR, CPP, JY and SJB wrote the paper.

## References for Supplemental Materials

Brenner S. 1974. The genetics of Caenorhabditis elegans. *Genetics* **77**: 71–94.

Friedland AE, Tzur YB, Esvelt KM, Colaiácovo MP, Church GM, Calarco JA. 2013. Heritable genome editing in C. elegans via a CRISPR-Cas9 system. *Nat Methods* **10**: 741–743.

Frøkjær-Jensen C, Wayne Davis M, Hopkins CE, Newman BJ, Thummel JM, Olesen S-P, Grunnet M, Jorgensen EM. 2008. Single-copy insertion of transgenes in Caenorhabditis elegans. *Nat Genet* **40**: 1375–1383.

Heger A, Holm L. 2000. Rapid automatic detection and alignment of repeats in protein sequences. *Proteins* **41**: 224–237.

Kadandale P, Chatterjee I, Singson A. 2009. Germline transformation of Caenorhabditis elegans by injection. *Methods Mol Biol Clifton NJ* **518**: 123–133.

Meneely PM, McGovern OL, Heinis FI, Yanowitz JL. 2012. Crossover distribution and frequency are regulated by him-5 in Caenorhabditis elegans. *Genetics* **190**: 1251–1266.

Mets DG, Meyer BJ. 2009. Condensins regulate meiotic DNA break distribution, thus crossover frequency, by controlling chromosome structure. *Cell* **139**: 73–86.

Rose AM, O’Neil NJ, Bilenky M, Butterfield YS, Malhis N, Flibotte S, Jones MR, Marra M, Baillie DL, Jones SJM. 2010. Genomic sequence of a mutant strain of Caenorhabditis elegans with an altered recombination pattern. *BMC Genomics* **11**: 131.

Wicks SR, Yeh RT, Gish WR, Waterston RH, Plasterk RH. 2001. Rapid gene mapping in Caenorhabditis elegans using a high density polymorphism map. *Nat Genet* **28**: 160–164.

Zetka MC, Rose AM. 1995. Mutant rec-1 eliminates the meiotic pattern of crossing over in Caenorhabditis elegans. *Genetics* **141**: 1339–1349.

## Supplemental Figures and Tables

### Supplemental Table 1. *C. elegans* strains used for this study.

### Supplemental Table 2. Primers and restriction enzymes used for genotyping N2 (Bristol) and CB4856 (Hawaiian) alleles in recombination assays.

### Supplemental Figure 1. Mutations in *rec-1* alter recombination frequencies in the *unc-101* – *unc-54* interval.

The targeted deletion of *y18h1a.7/rec-1 (h2875)* fails to complement the decreased recombination frequency in the *unc-101* – *unc-54* genetic interval. *The first allele indicates the homolog bearing the *unc-101* and *unc-54* mutations. Error bars indicate 95% CI.

### Supplemental Figure 2. Meiotic crossover events are redistributed in *rec-1(h2875)* mutant oocytes.

The redistribution of crossover events with no double-crossover has previously been observed in *rec-1(s180)* homozygotes using two-point mapping in hermaphrodites(Zetka and Rose 1995). (*A*) Oocyte genotypes derived from Bristol/Hawaiian hybrid animals in *rec-1(+)* and *rec-1(h2875)* genetic backgrounds. Strict crossover interference is observed in both genetic backgrounds in the interval defined by marker A* and E. *B* = Bristol allele, *H* = Hawaiian allele. (*B*) The *h2875* mutation distorts the oocyte genetic map, shown here with distances represented in map units (100 × [recombination frequency]). While the overall frequencies of recombination between markers A* and E and those between markers D and E are not significantly different between the two genetic backgrounds (*χ*^2^-test, *p* = 0.525 and 0.721 respectively), the individual intervals A* to B, B to C and C to D have significantly different recombination frequencies (*χ*^2^-test, *p* = 0.0105, 0.0389 and 0.0389 respectively).

**Supplemental Figure 3.** Alignment of *C. elegans* REC-1 and the putative *C. remanei* ortholog shows sequence similarity at the N termini of these predicted translations.

### **Supplemental Figure 4.** Day-by-day hatching and male progeny frequencies in *rec-1; him-5* double and transgenic strains.

(*A*) Hatching frequencies are exacerbated by maternal age, as similarly described by Meneely *et al.* (Meneely et al. 2012). Data from Figure 4a are shown separated out by age. Due to the low numbers of eggs laid after day 3, the tail end of the hatching period was grouped together (Day 3+). Kruskal-Wallace test for significance: **p* < 0.02, ***p* < 0.005, ****p* < 0.0001. Number of embryos counted is the same as Fig. 4a. (*B*) Frequencies of males in the populations of hatched progeny from (*A*). *rec-1* transgenes did not reduce the percentage of males in *rec-1 him-5* doubles. Numbers above or on the bars represent the number of adult animals counted.

### **Supplemental Figure 5.** Pairing and synapsis are unaltered in *rec-1* and *rec-1; him-5* double mutants.

Day 1 adult gonads of wild type, *rec-1(h2875), him-5(e1490),* and *rec-1(h2875); him-5(e1490)* double mutants were dissected and co-stained with DAPI to visualize DNA (green), anti-HIM-8 (yellow) to visualize the X chromosome pairing centers, and anti-SYP-1 (magenta) to visualize the synaptonemal complex. The presence of a single HIM-8 focus throughout the pachytene indicated complete pairing in wild type and mutants. SYP-1 begins to load in the transition zone and full synapsis is achieved prior to entry into early pachytene. These dynamics are unchanged by loss of *rec-1* or *him-5* functions.

### **Supplemental Figure 6.** RAD-51 foci numbers are reduced and distributed differently in *rec-1; him-5* double mutants

Day 1 adult gonads of wild type, *rec-1(h2875), him-5(e1490)* and *rec-1(h285); him-5(e1490)* were dissected and co-stained with anti-RAD-51 (magenta) and DAPI (green). The numbers of RAD-51 foci per nucleus were counted using 3-D image analysis and are shown in Fig. 4C.

### Supplemental Table 1. *C. elegans* strains used for this study.

| Strain name | Genotype | Description |
| --- | --- | --- |
| VC2010 | +, Bristol | Bristol N2 wild type sub-culture kept at the Moerman Gene Knockout Lab at the University of British Columbia. |
| CB4865 | +, Hawaiian | Hawaiian wild type sub-culture archived at the Caenorhabditis Genetics Center. |
| BC313 | *rec-1(s180)* I | *rec-1(s180)* mutation (Q29 > STOP) |
| KR5301 | *rec-1(h2872)* I | *rec-1(h2875)* mutation, a 1-bp deletion in exon 2. Not outcrossed after CRISPR-Cas9 mutagenesis. |
| KR5305 | *rec-1(h2875)* I | *rec-1(h2875)* mutation, a 64-bp deletion in exon 2. 6 × outcrossed to VC2010 after CRISPR-Cas9 mutagenesis |
| KR5306 | *rec-1(h2875)* I, Hawaiian | *rec-1(h2875)* mutation, a 64-bp deletion in exon 2. 6 × outcrossed to CB4865 after CRISPR-Cas9 mutagenesis |
| EG6699 | *ttTi5605* II; *unc-119(ed3)* III; *oxEx1578* | *Mos*I insertion on Chromosome II (*ttTi5605*) with the *unc-119* mutation balanced by *oxEx1578*. Unc-119 segregants used for *Mos*SCI experiments |
| DW687 | *dwSi4[rec-1(+) Cbr-unc-119(+)]* II; *unc-119(ed3)* III | Wild type *rec-1,* with its endogenous promoter and 3' region, inserted at the *ttTi5605* site. |
| KR5326 | *rec-1(h2875)* I; *dwSi4[rec-1(+) Cbr-unc-119(+)]* II | Derived from KR5305 and DW687 |
| DW688 | *dwSi5[rec-1(8S/T>E) Cbr-unc-119(+)]* II; *unc-119(ed3)* III | *rec-1(8S/T>E),* with the *rec-1* endogenous promoter and 3' region, inserted at the *ttTi5605* site. |
| KR5327 | *rec-1(h2875)* I*; dwSi5[rec-1(8S/T>E) Cbr-unc-119(+)]* II | Derived from KR5305 and DW688. |
| DW690 | *dwSi6[rec-1(8S/T>A) Cbr-unc-119(+)]* II; *unc-119(ed3)* III | *rec-1(8S/T>A),* with the *rec-1* endogenous promoter and 3' region, inserted at the *ttTi5605* site. |
| KR5328 | *rec-1(h2875)* I; *dwSi6[rec-1(8S/T>A) Cbr-unc-119(+)]* II | Derived from KR5305 and DW690 |
| CB4088 | *him-5(e1490)* V | *him-5(e1490)* mutation |
| RB1562 | *him-5(ok1896)* V | *him-5(ok1896)* mutation |
| QP833 | *rec-1(s180)* I; *him-5(ok1896)* V | Derived from BC313 and RB1562 |
| QP856 | *rec-1(h2875)* I; *him-5(ok1896)* V | Derived from KR5305 and RB1562 |
| QP857 | *rec-1(h2875)* I; *him-5(e1490)* V | Derived from KR5305 and CB4088 |
| QP962 | *rec-1(s180)* I; *him-5(e1490)* V | Derived from BC313 and CB4088 |

### Supplemental Table 2. Primers and restriction enzymes used for genotyping N2 (Bristol) and CB4856 (Hawaiian) alleles in recombination assays.

| Reaction | Primer name | Sequence | PCR product digested with |
| --- | --- | --- | --- |
| A* | IIIA-F  IIIA-R | gcataaaccggctaaaaatcg  ttgcagggtatatgacttctgg | *Aat*II – Digests only CB4856 allele |
| B | IIIB-F(new)  IIIB-R(new) | catcattttccacttctgaaacc  ctcctccgagaagctcaagg | *Spe*I – Digests only CB4856 allele |
| C | IIIC-F(new)  IIIC-R(new) | aaatcttgctgttccttgtcc  caccttaatcccttcaaaacg | *Acc*I – Digests only N2 allele |
| D | IIID-F(new)  IIID-R(new) | caccaatgttttccgcacagc  ttgatgatgcatttgattattgg | *Dra*I – Digests only CB4856 allele |
| E | IIIE*-F  IIIE*-R | tgggagaaaatcgaaaatcg  gcctaagcctatgcctatgcctaaacctaagcctaagcccGagctttagc | *Hin*dIII – Digests only CB4856 allele |
